# Some Recent Developments of the Germ Theory, More Particularly in Relation to the Treatment of Phthisis

**Published:** 1888-12

**Authors:** R. Shingleton Smith

**Affiliations:** Honorary Fellow of King's College, London; Senior Physician to the Bristol Royal Infirmary; and Lecturer on Medicine in the Bristol Medical School


THE BRISTOL

### Medico-Chirurgical Journal.

DECEMBER, 1888.

### SOME RECENT DEVELOPMENTS OF THE GERM THEORY,

### MORE PARTICULARLY IN RELATION TO THE TREATMENT OF PHTHISIS.

The Presidential Address, delivered on October 10th, 1888, at the opening of the 15th Session of the Bristol Medico-Chirurgical Society.

BY

R. SHINGLETON SMITH, M.D. Lond., B.Sc., F.R.C.P.;

*Honorary Fellow of King's College, London;*
*Senior Physician to the Bristol Royal Infirmary; and*
*Lecturer on Medicine in the Bristol Medical School.*

TWICE in the history of this Society have our presidents directed the attention of its members to the influence of germs in the production of disease. Our first president, Dr. Brittan, in the year 1874, propounded, illustrated, and illuminated this question: first, from what we know of diseased blood, and of the "blood diseases;" secondly, from what we know of the blood-germs, and bioplastic matter; whether, thirdly, there is not good ground for supposing these "blood diseases" to be due to morbid conditions of the blood-germs. Four years later, Dr.

Henry Fripp illustrated the doctrine of *contagium vivum* in its relation to parasitic disease, from which I quote the following paragraphs. He says: "The germ theory has to those who accept it such momentous issues, that the study of the life history of the germ becomes the *sine quâ non* of its acceptance as a living contagium. For if it be regarded as the determining cause of infective disease and of its specific character, the proof that it is an independent organism, which can live and maintain its typical development unaffected by its surroundings, and finally breed true, is necessary before any reliance can be placed in the supposition that invariability of the morbid phenomena and of their sequence, in a word, of the type of disease, is the direct consequence and visible expression of the phases of germ life. This implies that each low organism detected and charged with the crime of poisoning has no *alias*, no variety of reproductive form or phase in its life history, or else that it is contagious in one particular form."

Again, he adds: "From the above considerations, it appears that the germ theory represents, though in a more precise and material form than was possible in premicroscopic times, the long dormant doctrine of *contagium vivum*. The germ is said to be that actual concrete material entity, named when it was still but an abstract conception, *contagium*, on account of its observed and experienced effects. It embodies the hypothesis of parasitic contagium, and justifies the predicate *vivum*."

It was from our own city, although our Society is not old enough to claim its author, that, in 1867, the doctrine of the contagiousness of phthisis was first announced to the world by Dr. William Budd,* whose name should also

* *Lancet*, Vol. II. 1867.

be inseparably associated with the doctrine of the propagation of fevers by "fever seed."

I wish this evening to illustrate this question still further, in the light of discoveries which are now universally admitted to be intimately connected with the cause of phthisis. My immediate predecessor, in that eloquent address of last year which we all remember, remarked that "our hospitals are not affording us the information we have a right to expect from them." Accordingly, on thinking over a subject for this evening, I decided to attempt an outline of the various attempts we have made in our hospital work towards elucidating the problems of treatment associated with the germ theory of phthisis. Professor Jaccoud, in 1884, asserted with emphasis that the germ theory had as yet done nothing for phthisical patients. We may now not unprofitably enquire whether it has not done, and is not doing, much which may be of benefit, even although the results may often appear to be disappointing and unprofitable.

One great fact is an obvious outcome of the conclusion that, if the bacillus is the *materies morbi*, the *contagium vivum*, we no longer doubt the possibility of the dissemination of the disease by means of particulate germs, such as are believed to exist in all contagious diseases, and which are now proved for phthisis. This is no new idea, but it is one which has been actively combated, and even in recent years denied, because of the limited manifestations of contagion which are commonly met with. We may now consider that this question of contagion is definitely settled; for, if a local tuberculosis commonly results from the inoculation of a pure cultivation of the bacillus, and does not arise from anything from which the bacillus is certainly excluded, what possible escape can we find from the conclusion that the disease resulting from the presence of this contagion may be disseminated as other contagious diseases are?

The resolutions adopted by the Congress for the study of tuberculosis at its recent meeting in Paris, to which you did me the honour to appoint me as your delegate, afford the best possible proof of the unanimity of opinion on this question, inasmuch as the adoption as an axiom of the contagious nature of tuberculosis was made the foundation of decisive action for the prevention of the disease by legislative regulations, such as are enforced in the case of epidemic and other contagious diseases in animals. The decree, by which this law came into operation in France, was signed by the President of the Republic during the session of the Congress; and here we, doubtless, have one of the most important epochs in the history of the disease.

We do not doubt the possibility of contagion of typhoid, nor do we that of syphilis. All the arguments which have been used against the contagiousness of phthisis may be applied with almost equal force to either or both of these affections. Further, the question of leprosy has been often discussed, and opinions have varied just as they have with phthisis. Now that a bacillus of leprosy is an admitted fact, and a bacillus of syphilis a probable one, may we not class these affections together, and reason by analogy from one to the other? The arguments with regard to the contagion of leprosy and tuberculosis appear to have been almost identical, and have chiefly been founded on the fact that the disease has not spread in either leper or consumption hospitals. Is it not probable that the admission of the possibility of contagion in one case as in the other may be the beginning of a new era in the history of both?

It has been said* that "the civilised world, medical and lay, is rather apathetic about consumption. It has gotten rid of the plague, and nearly rid of typhus epidemics; leprosy has been driven out of England, and smallpox has been made manageable: but one death in seven from all causes is still due to *tuberculosis pulmonum*, and some part of the remainder is due to other tuberculous diseases. If we feared these diseases as they merit, as we do the cholera or yellow-fever, we would in time suffer less from their ravages. But we have strangely grown used to them, and view them with a sort of fatalistic indifference, broken now and then by a ripple of interest awakened by the discovery of some new fetish—a wash-bottle, or an air-tight box, or some ingenious device, the impotent offspring of mechanical skill and ignorance of pathology."

A writer in the current number of the *Medical Chronicle* remarks that "Disinfection, or, in other words, the destruction of the *contagium vivum*, is the most effective means of combating the infectious diseases. The shortcomings of this means of treatment do not spring from a want of methods, but from our deficient knowledge of the exact nature of the contagia in the different diseases, and because we do not yet know how those contagia are affected by the various physical and chemical agents proposed for their destruction." Even methods which, from experiments in the laboratory, appear to be perfect, fail when adopted in practice. The same means which will be effectual with one kind of contagium, will be inert in another. A recent publication † advocates the biniodide

* *International Journal*, October, 1888.

† *The Abortive Treatment of Specific Febrile Diseases by the Biniodide of Mercury*. Illingworth. Lewis, 1888.

of mercury in doses of $\frac{1}{16}$, $\frac{1}{8}$, or $\frac{1}{4}$ grain three times a day, and the author has no doubt as to its efficacy in small-pox, typhus, plague, relapsing fever, dengue, yellow fever, hydrophobia, glanders, and leprosy; because he has successfully treated in this way scarlet fever, diphtheria, measles, chicken-pox, whooping-cough, mumps, enteric fever, pyæmia, puerperal fever, and syphilis.

Now, tuberculosis is almost the only specific contagious disease excluded from this list; but we fail to see why it should be so excluded from the range of operation of this omnipotent germ-slayer. Other authors have attempted to utilise this drug in the treatment of phthisis, and we shall see with what success hereafter.

Utopian as it seems to be to attempt to find a universal remedy against all kinds of specific diseases, and indisposed as we are to accept evidence such as the above, where bald statement takes the place of proof, and unverified inference the place of experience, we believe that with increased knowledge, derived by experiment on each kind of germ product, means of disinfection may possibly be found for each of the members of the group, which may give results similar to the effects of mercury in syphilis, or quinine in ague.

It being conceded that the essential condition underlying the phenomena of phthisis is the *bacillus tuberculosis*, it naturally follows that the destruction of the parasite should be attempted, either by what has been well named the offensive method, the direct action of germicides, or by the defensive method—the rendering the soil unsuitable for its existence. The problem as regards the lung is a complicated one: both lungs being often implicated, or the different lobes of one lung, a large area is involved, and the diseased patch can only be reached efficiently by the blood-channels. If the lung were a simple cavity, and could be treated as a cold abscess is, by injection, the problem would be comparatively simple: it would then resolve itself into the treatment of a local tuberculosis. It will, then, be well to enquire, first of all, as to the phenomena of a local tuberculosis and its local treatment, before we attempt to deal with the more complex conditions attending diffuse tubercular phthisis.

Now, the best possible illustration of a local tuberculosis is that produced by experimental inoculation in the rabbit's eye, where the different stages of the disease can be gradually watched from the local development of the bacilli up to their gradual dissemination throughout the body by blood- and lymph-channels. These changes have been recently well described in detail by Mr. Barker, in his lectures at the Royal College of Surgeons, published in the *British Medical Journal* (June 9th, 1888), and need not detain us further.

Such inoculations are now well recognised as having frequently occurred in the human subject. Many such are recorded. A series of them is briefly detailed in the *British Medical Journal* of June 2nd, 1888.

A still more remarkable series of cases has been recorded by Lehmann, and quoted by Barker, in which ten children were inoculated into the prepuce from the mouth of the operator, who was dying of tubercular disease of the lungs.

Another striking case, also quoted by Barker, is that of a veterinary surgeon who punctured the joint of his left thumb whilst dissecting a tubercular cow, and who died of bacillary disease in the lungs a year and a half afterwards.

Numerous cases of tuberculosis of the urinary tract have been recorded by Cornil and others,* in which numerous bacilli have been found in the urine. I have myself met with several cases of this kind, one of which, recorded in the *Lancet* (Vol. I. 1883, p. 942), was one of the first in which bacilli had been found in the urine, and was an excellent illustration of tuberculosis beginning in the urinary tract, and limited to it for several years, but becoming afterwards disseminated throughout both lungs in a very virulent and active form. This and some other cases have enforced upon me the necessity for and utility of the examination of urine for bacilli in obscure cases of vesical and renal diseases, where their presence leaves one in no doubt as to the gravity of the disorder; and, on the other hand, their absence enables us to put aside the probability of strumous pyelitis and tubercular cystitis.

Cases of local tuberculosis of skin have been recorded. Babes and Cornil mention three, in which bacilli were found in the tissues diseased. The cases were:

1. Perineal ulcer in a phthisical man.
2. Ulceration of the lower lip of a phthisical woman.
3. Tuberculosis of the vagina in a tubercular subject.

These cases naturally are associated with the questions as to the nature and treatment of lupus, a disease which commonly has been more associated with syphilis than with tuberculosis, but which has now been shown to be essentially tuberculous in its nature, although not often accompanied by or leading to acute tubercular disease of the lungs or elsewhere. The fact that tubercle bacilli may be found in lupus tubercles, of necessity suggests the importance of active treatment, by which the diseased tissue may be destroyed whilst it is strictly local; and,

* *London Medical Record*, November, 1883.

on the other hand, the rarity of dissemination of this disease illustrates the importance of the suitability of soil in which bacilli are implanted, in order that they may germinate actively and become disseminated: it is interesting to observe that the principal methods of medicinal treatment in vogue, cod-liver oil and arsenic, are just those which might be expected to render the soil unsuitable.

Another form of local tuberculosis of considerable interest to the surgeons is that of the bones and joints. It has now been clearly shown that there is no difference between tubercular and scrofulous diseases of bones;* the characteristic giant cells in association with lymph-cells, and the presence of bacilli, which, although difficult to find, have been found by Koch and other observers. These facts, confirmed by the production of artificial tuberculosis by inoculation of scrofulous material, stamp the scrofulous process as a tubercular one, and enable us to illustrate the general disease from the phenomena of the local one. It is not a little remarkable that the bodies of the vertebræ should be the special habitat of the bacilli; and the question how the parasite finds its way to the cancellous texture of vertebræ or short bones, and should be limited to this one spot in the body, is extremely difficult to answer. As pointed out by Barker,† the pia mater of the child and the medulla of the vertebræ are both removed from external influences, and yet these are the seats not uncommonly chosen for the first manifestation of the presence of tubercle bacilli within the body: he infers that there must be some inherent suitability of soil in certain spots—that, in fact, some tissues are, by

* *Diseases of Bones*. Thomas Jones. p. 175. 1887.

† *British Medical Journal*, June 9th, 1888.

hereditary predisposition, a fertile soil ready for the growth of the germs, if by any means these happen to be engrafted or introduced, either by blood- or lymph-channels. Again, it is of interest to note that a local tuberculosis, even of bone or joint, does not necessarily lead to fatal infection and dissemination. Barker mentions the case of a boy of twelve years of age, operated on thirty-five times in seven years, and cured of tuberculous bone disease at last.

On the other hand, the risk must always be a considerable one, more especially where spinal abscesses are in contact with lung; as in a case of my own, where Mr. Greig Smith aspirated several times a localised empyema resulting from spinal disease, which led ere long to the development of active bacillary phthisis in both lungs.

Dr. Gerster,* of New York, points out an interesting fact in connection with the treatment of tuberculous joint affections. He says that the progress is frequently retarded, or even completely arrested, by the prolonged application of cold in the form of an ice-bag to the affected joint, and that this is in accord with Koch's observations on the growth of the bacillus, which requires a considerable temperature for germinal activity. The spores are not killed by cold, but their propagation is arrested; and in this way time is allowed for strengthening the power of resistance of the organism, and healthy tissues may defend themselves by a protective encapsulating wall of granulation tissue.

Local tuberculosis of joints is to be treated, according to Gerster, by the early finding out and evacuation of tubercular cheesy foci in the epiphysis of the bones before

* *Annals of Surgery*, April, 1888.

the disease has extended to the joint, and by early excision of hip, knee, or ankle, when the joint has become affected; it is necessary to conscientiously remove all the tissues, soft or osseous, which are found to be manifestly diseased. He says "we have to deal with tuberculous tissues as though they were cancerous," and he gives a caution against the possibility, shown by Koenig, of the operative dissemination of tuberculosis, *i.e.* infection by dissemination of the virus from the wound into the circulation by means of the mechanical effects of the operation itself.

Destruction of the part involved is also the keystone of treatment as laid down by Jones of Manchester; but he insists also on constitutional treatment, adding that the prognosis is never hopeless provided there be good food and plenty of fresh air.

Another kind of primarily local tuberculosis is that beginning in the alimentary canal as a result of food contamination, of which the following is one of the most notorious examples, related by Herterich.* A phthisical mother had two little girls, aged respectively fifteen and three months. These children throve well so long as they were suckled; but when they were weaned, their mother used to feed them by chewing their food partially herself, and then putting it in their mouths. For a time all went well; but as her expectoration increased, both children, as a consequence of this disgusting practice, began to emaciate, and died with tubercle in various organs, the mother surviving the children a few months.

The observations of Mosler† on intestinal tuberculosis from swallowing sputa; the presence of tuberculous ulcers

* *Birmingham Medical Review*, March, 1884, p. 141.

† *London Med. Record*, November, 1883.

of the palate, with bacilli, as shown by Guttmann*; the occurrence of tuberculous sloughing of the tonsils, with bacilli in the *débris*, as I have myself observed; the presence of bacilli in abscesses near the anus, in phthisical subjects†; the finding of bacilli in the fæcal evacuation in cases of tuberculous diarrhœa, are illustrations that in the alimentary canal the process is the same as elsewhere, and that bacillary inoculation, growth, and development are the *sine quâ non* of the disease. It has recently been shown by Cornil‡ that penetration by simple contact with the mucous membrane is possible, without any evident lesion; that after an ingestion of some drops of culture into the intestine, bacilli may be found in the mesenteric glands after five or six days. The observations of Chauveau, Villemin, Parrot, and Cornil, have abundantly proved this for the intestinal tract, as also for the pulmonary and vaginal mucous membrane; and it has recently been shown by Arloing§ that, after infection by alimentary canal, it is impossible to arrest the progress of experimental tuberculosis, inasmuch as extirpation of the affected glands was found to be useless. On the other hand, it has been pointed out by Coats‖ that tuberculosis is not usually a penetrating lesion, but that it rather extends on the surface of membranes and along canals or other channels: this is remarkably the case with regard to tuberculosis of the intestine on the one hand, and of the peritoneum on the other. "There is scarcely any tendency to extension of intestinal tuberculosis to the peritoneum generally." "An interesting corollary to this is the converse con-

* *London Med. Record*, November, 1883. † *Lancet*, I. 1883, p. 1108.
‡ *Le Progrès medical*, August 4th, 1888.
§ *Ibid.* ‖ *Lectures to Practitioners.* Longmans, 1888.

dition, in which the peritoneum is the seat of tuberculosis, while the intestine is not affected. Tubercular peritonitis is a superficial tuberculosis of the peritoneum as a whole; but the tubercles apparently confine themselves to that membrane, and do not readily penetrate into the wall of the intestine." "All observers agree that, in at least half of the cases which die of phthisis pulmonalis, tubercular ulceration of the intestine is present. The lesion is due to tubercular material being carried into the stomach and intestine, instead of being discharged by the mouth."

Few attempts have been made to disinfect the alimentary canal, and so to prevent auto-inoculation by this route. The use of carbolic acid, with or without tincture of iodine, according to the method of Rothe in typhoid, and well reported of by Gramshaw (*Lancet*, 1888, I., 1243), might be of service in this respect. In one case I have used the $\beta$-naphthol with fairly good result, the patient taking the drug easily in sufficient quantity.

The very numerous experimental researches in inhalation tuberculosis, show the way in which the disease arises locally along the respiratory tract; the injection of pulverised tuberculous sputa into the trachea, by means of a spray producer, giving rise to tubercular changes in the lungs in dogs and rabbits, whereas the injection of non-tuberculous material gave no such result (*London Med. Record*, Nov. 1883, p. 475 and 477).

According to Coats, "The disease begins in the finer bronchial tubes. The focus of the disease in each little centre is an inflamed and tubercular bronchiole." "The bronchioles being the primary seat, we shall find that there are two open channels by which the infection may extend; namely, the air-passages and the lymphatics." The mucous membrane of larger tubes becomes eroded; exposure and necrosis of the cartilages in the bronchi and trachea occur, as they do also in the larynx, but the smaller bronchi are more affected than the large ones. Cases of general dissemination from auto-inoculation, from bronchial gland, from larynx, and from wall of pulmonary vein, are described by Coats.

The illustrations I have given afford us abundant evidence that, in the great majority of cases, tuberculosis is at the beginning the result of a local infection or inoculation from outside the body: even in the case of the lung it is commonly only one lobe, and even for years the disease may continue to be limited to one lung or even one lobe only. We have at this stage to deal with a local tuberculosis, and the question naturally arises, whether it may not be possible to treat the disease, whilst it is local, in accordance with the same principles which are brought into operation in the case of the other forms of localised tuberculosis to which I have alluded, and with equal success. In accordance with this view, the method of treatment by parenchymatous injection has been attempted, and this it is to which I wish to direct your especial consideration. There are, however, three principal methods by which a focus of disease may be reached medicinally:

*a.* By the introduction of substances into the blood of the body as a whole.

*b.* By parenchymatous injections into the part affected.

*c.* By inhalations, and pulverisations along the air-tubes.

The first method—that of general medication—includes all the ordinary medicinal substances which have had a reputation for utility when given by the alimentary canal, and also the hypodermic method. These two methods must detain us awhile, as they are a necessary antecedent to the more strictly local measures, by which the substances are caused to reach the part affected, whether it be by injection through the chest-wall, or by insufflation along air-tubes.

My observations in the direction of the general antiseptic treatment of phthisis by internal medication, have been almost entirely limited to two sets of drugs: A. Iodoform and iodol; B. Creasote and guaiacol.

A. The utility of iodoform in lung disease was first pointed out by Semmola, at the International Congress meeting in Amsterdam, in 1878. The cases treated were mostly bronchial catarrh, asthma, broncho-pneumonia. He disclaimed any intention of bringing forward iodoform as a remedy for tuberculosis, or all caseating affections of the lung; but he found that cases of incipient phthisis were cured, and even in advanced cases expectoration is lessened, cough and fever are moderated, caseation is arrested, and life is prolonged. The expectation that the germicide action of the drug might be of service led Dr. Dreschfeld, of Manchester, to administer the drug in a large number of cases, and he claimed the following benefits from its use: increase of weight and appetite, with diminution of temperature, night-sweats, cough and expectoration. At the International Congress of Copenhagen, in 1884, I gave an analysis of forty-six cases, with tables showing variations in weight before and after treatment. An examination of these tables shows that twenty-nine out of the forty-six patients manifested an absolute gain in body-weight; of the remaining seventeen the loss of weight was small, and in many of those the wasting, which had been rapid, was more or less completely arrested.

Since the publication of this paper I have continued the use of iodoform, either in pills or in solution in cod-liver oil, and have had abundant reason to believe in its utility. I regret that the difficulties of getting accurate statistics from out-patients, and the want of time to record and analyse the results of in-patient and home practice, prevent me from giving further mathematical demonstration of the value of the drug. I am well aware of the difficulties associated with the question as to the effects of any drug in such a disease as phthisis, and the fallacies of inferences from a small number of cases observed for only short periods. An excellent illustration of such fallacies is, I believe, to be easily obtained, even with regard to this very drug, which has been asserted to be an immediate and infallible hæmostatic in cases of hæmoptysis.*

Current medical literature teems with illustrations of the satisfactory results to be obtained from iodoform, both in local and general tuberculosis, although the germicide value of the drug has been so frequently called in question; but, as regards its hæmostatic power, several of my cases have had abundant and dangerous hæmorrhage whilst taking the drug in full doses, and after a prolonged use of it: hence I cannot accept the statement that small doses of it will arrest hæmorrhage. The fear of toxic results must have been much exaggerated. Its use in large doses diluted with charcoal, in typhoid fever, for the purpose of disinfecting the alimentary canal, shows it to be a harmless drug in the great majority of cases. The physiological limit of dose probably varies much with the individual case—even small doses irritate some patients; but its insolubility probably prevents any toxic action,

* Chauvin et Jorissenne. *Le prog. Med.*, May 19th, 1888.

even when given to others in larger quantity.* In the great majority of cases there is no idiosyncrasy, and the iodoform agrees well in doses of one grain or more, and may be continued for many months without fear of any toxic results. The combination with terebene, or creasote, either in capsules or in the cod-liver oil, gives us an opportunity of varying the form of administration from time to time, according to the necessities of the case.

Time fails me to allude to the use of iodoform as a local application for tuberculous diseases. As an inunction with lanoline it has been reported to give good results in cases of tubercular meningitis; and still more recently this disease is said to have been treated successfully by iodoform given internally.†

Advantages have been claimed for iodol over iodoform; but I have not found them to be of great value from the medical standpoint, excepting on the ground of greater solubility in ether. This substance, tetra-iodo-pyrrol, $C_4I_4NH$, containing 89 per cent. of iodine, whereas iodoform, $CHI_3$, has 96 per cent., being free from odour and taste, may for surgical purposes have advantages which will more than compensate for the slightly smaller proportion of iodine; but for internal use I have never found the odour or taste to be any drawback to the use of iodoform, inasmuch as with coated pills and in cod-liver oil both are effectively concealed; and, as a matter of experience, I have thought that the iodol is not equal in therapeutic value to the other. From both drugs the iodine is easily liberated within the body, and appears in the urine as an alkaline iodide: they are therefore

* *Vide* Transactions of International Congress, Copenhagen, 1886.

† *Le Prog. med.*, 11th August, 1888.

broken up within the body. But it has been shown by Professor Pick, of Prague, that their elimination is much more gradual than is the case with the potassic iodide, traces of iodine being found in the urine five days after the cessation of iodol, but only two days after the cessation of the iodides. The greater solubility of the iodol gives it an advantage for purposes of injection, and I have used it frequently for this purpose: it does not appear to be either more or less irritating to the cellular tissue than iodoform; but inasmuch as both of these drugs usually give rise to some local irritation, I have for the present discarded them in favour of others, which appear to involve less risk of damage to lung, whilst they are likely to be equally effectual in modifying the local pathological process.

B. Creasote has had a considerable reputation in the treatment of lung diseases;* but it is only recently that it has begun to take its place as an efficient germicide in phthisis. The publication of the results obtained by Gimbert and Bouckhardt of ten years ago, and of Sommerbrodt in 1887, has given an impetus in this direction, as tending to show that the value of the drug is in direct proportion to the size of the dose, and that large doses will succeed where small ones have failed, as was the case with Bogdanovitch, † who took five grains four times daily in capsules after food with great benefit, whereas he had previously found that small doses were useless.

I have found that patients readily take it in combination with bitter tinctures and glycerine or cod liver oil; many of my out-patients are taking some such combination, and are progressing favourably under its use.

* *Medical Chronicle*, Oct., 1888. † *Brit. Med. J.*, March 3rd, 1888.

The nauseous taste and irritating properties of creasote have led to the substitution for it of the substance guaiacol, a colourless liquid, having the composition of catechol monomethyl ether, $C_6 H_4 \begin{cases} OH \\ OCH_3 \end{cases}$, and contained in beechwood creasote to the amount of 60 to 90 per cent. Sahli of Berne was led to prefer it to creasote because of its more definite composition, and less unpleasant odour and taste. He prescribed it thus:

℞ Guaiacol puriss. ... ... ♏ 15 to 30

Aq. dest. ... ... ... ℥vj.

Sp. Vini Rect. ... ... ʒvj.

A teaspoonful to a tablespoonful, two or three times a day, after food, in some water.

Fræntzel* considers it the active constituent of creasote, and strongly advocates the following formula:

℞ Guaiacol ... ... ... ʒiiiss.

Træ. Gentian. Co. ... ℥j.

Sp. Vini Rect. ... ... ℥viij.

Vini Xerici q. s. ad Oj.

One tablespoonful, two or three times daily, in a wineglassful of water.

Berlin physicians have given these drugs in gelatinous capsules, and the French physicians have administered them in the well-known "pearls" of creasote and iodoform of Dr. Clertan, in which the more active drugs are dissolved in ether, with the object of facilitating the absorption, diffusion, and transport of the drugs to the lungs, by which route they may be eliminated. In this way creasote can be administered for long periods without inconvenience: Sommerbrodt's patients continued it for several months, and some patients had taken six to twelve hundred capsules

* *Med. Chronicle*, Sept., 1888.

without interruption. I have been in the habit of prescribing capsules containing five grains of creasote in combination with one grain of iodoform, and have not met with any inconvenience or unsatisfactory result from their use, beyond their relative expense in comparison with the solutions in spirit.

Internal administration of drugs by means of rectal and hypodermic injection has been much in vogue of late, and our experience of these two methods will not be devoid of interest.

Bergeon's device of gaseous injections into rectum was tested by myself and colleagues immediately after his results were first announced, but has long ago been discontinued. I have here a record of two cases in which the injections were made with regularity, the carbonic acid being made to bubble through the eaux bonnes, and then to pass into the rectum in quantity amounting to about four litres daily. There was no difficulty in carrying out the process, but it had no effect whatever on the course of the disease or in alleviation of symptoms. This result appears to be pretty much that met with by other observers; and hence the "Bergeon fizzle" is now dead and buried, and the apparatus consigned to the lumber-room.

The hypodermic method, on the other hand, is one which is rapidly becoming more popular, and which seems likely to be of some service in conjunction with other measures. It is argued that the digestive tube is defective in consumptive patients, and that, accordingly, the skin is the best means for the introduction of substances into the blood in sufficient quantity to reach and modify the spot or spots in which the lesions may exist. Various substances and various vehicles for them have been tried, and have met with a measure of success. Carbolic acid has been used by Dieulafoy and Dujardin-Beaumetz,* Lefaivre,† Filleau and Leon Petit ; ‡ but eucalyptol, iodoform, iodine, arsenic, and salicylic acid have also been employed in this way. One of the best diluents for the purpose of hypodermic injection was that suggested by Meunier, of Lyons § —liquid vaseline.

All the various methods of treatment by hypodermic injection are obviously only modifications of general medication, the drug being introduced into the cellular tissue of any part of the body, commonly a healthy part, in order that it may reach the blood and be carried in the blood-stream to the part diseased. It follows that, to be of service, the drug must be introduced in sufficient quantity that the whole mass of blood in the body may have such a percentage as is likely to modify the germ development in the part affected ; and the question naturally presents itself, whether it is possible to introduce, either by stomach or by cellular tissue, a sufficient quantity of any potent germicide to influence a diseased focus, without at the same time so poisoning the whole mass of blood as to more than counteract any possible benefit to be obtained. It may be possible to accomplish the end in view, but it is clear that the various methods of hypodermic injection now in use are altogether futile, inasmuch as the quantities introduced are entirely inadequate when distributed throughout the whole mass of the body. It was pointed out by Sternberg,|| some years ago, that the quantity of iodine required to prevent the development of septic test organisms is one part in four thousand. It follows that the quantity of iodine necessary to prevent

* *Medical Recorder*, March, 1888.

† *Gazette des hôpitaux*, May 12th, 1887, p. 471. ‡ *Bull. de la Phth. Pulm.*, No. 4.

§ *Bull. Gen. de Thérapeutique*, Jan. 15th, 1887.

|| *American Journal of Med. Sc.*, April, 1883.

the development of the septic micrococci in the blood of an adult man weighing 160 pounds would be 35 grains—a dose of iodine which it is entirely impracticable to administer; but as iodoform contains 96 per cent. of iodine, and as I have found it possible to administer 30 grains daily for a whole month continuously before toxic symptoms were developed, we have here a quantity introduced within twenty-four hours which approximates the amount of iodine required in the blood at any one time to prevent germ development. Accordingly, it is not altogether a ridiculous idea to endeavour to find a germicide which the blood will tolerate in sufficient quantity for the manifestation of germicidal powers.

Until this problem has been satisfactorily solved, one cannot but consider the hypodermic method of general medication to be a somewhat fallacious plan. Clinically, it may appear to give good results; but it cannot rest on a good scientific basis so long as such infinitesimal and inadequate doses are relied upon to effect what appear to be almost impossibilities.

*b.* Failing the further and more satisfactory development of the hypodermic method, numerous attempts have been made to accomplish by local parenchymatous intra-pulmonary injections a more active and effectual attack on one or more diseased foci. I do not here allude to the subject of the injection and drainage of pulmonary cavities; this is an entirely distinct, and for the most part a surgical question, which is beyond my province; but in speaking of parenchymatous injections into lung, I refer to the diffusion of a fluid throughout a diseased patch of lung, commonly more or less solid lung, on the principle of local treatment of a localised tuberculous process.

Such injections have been frequently performed by

Pepper of Philadelphia, Beverley Robinson* and White* of New York, Ransom† of Manchester, and many others. One of the most remarkable series of these is that of Professor Riva,‡ of Pavia, who endeavoured to inundate the parenchyma of the lung so thoroughly with the fluid as to destroy all the bacilli present; as much as 40 to 50 c.c. of a 1 to 3000 solution of mercurial bichloride was injected at one time, and in upwards of one hundred injections no accident occurred. The number of bacilli in the sputum notably diminished, but in no case did they entirely disappear. Our own experience at the Bristol Royal Infirmary is now considerable. My first series of cases was published in the *British Medical Journal*, October, 1886; and since that time injections have been frequently performed, although with only partial success. The first substance I employed for injection was iodoform, and for two reasons—that it had been found to be of service when given by the alimentary canal, and that it appeared to be the most active germicide which could be safely introduced in sufficient quantity.

The solubility of iodoform is as follows:

| | |
|---|---|
| Absolute Ether ... ... | 1 in 8 |
| Rectified Spirit ... ... | 1 in 80 |
| Oil of Eucalyptus ... ... | 1 in 8 |
| Oil of Almonds ... ... | 1 in 60 |
| Olive Oil ... ... ... ... | |
| Vaseline Oil ... ... ... | |

The solution in oil was soon set aside, because of the supposed risk of fat-embolism, and that in ether was adopted. This has only the disadvantage that, by causing temporary giddiness and head disturbance, the patient

* *New York Med. Record*, 1886. † *Med. Chronicle*, January, 1887.

‡ *New York Med. Record*, April 23rd, 1887.

becomes timid of the injection, and unwilling to have it repeated. The oil of eucalyptus was then chosen as the solvent; but in two cases it gave rise to so much pain and extensive pleuritis, that neither patient would have consented to the repetition of the process: accordingly, this solution was entirely discarded, and has not been again employed. The quantity of solution injected has usually not exceeded one grain of iodoform, it being thought that the local action of this quantity in the local patch of tissue injected might be sufficient.

The following case illustrates the use of iodoform injected into the lung, and the subsequent course of the case:

Abraham L., æt. 30, chronic bacillary phthisis, had been under treatment for eighteen months as out-patient, from December, 1883, to July, 1885, and had taken iodoform during the whole period. Weight had increased from 8 st. 2 lbs., in December, 1883, to 8 st. 9 lbs., in September, 1884, after which time it remained stationary till May, 1885, when he began to lose weight, and had other symptoms of more active disease in the left upper lobe. Five injections of ethereal solution of iodoform were made into the consolidated patch during a period of ten days, at the supra-clavicular, infra-clavicular, and superior axillary regions. The first injection of ten minims of ethereal solution, containing two grains of iodoform, gave rise to cough, with momentary faintness; the following injections were limited to five minims, but some pain and a localised pleuritic friction followed the third; the fifth was followed by neuralgic pain in the shoulder and up the neck. The temperature continued to be subnormal, and the weight stationary. He was made out-patient in July, 1885, weighing 8 st. 4 lbs., and has continued to attend regularly ever since. The following is a tabulated summary of the history to the present time, indicating the different kinds of treatment adopted:

| DATE. | WEIGHT. st. lbs. | | TREATMENT. | REMARKS. |
|---|---|---|---|---|
| 1885—Aug. 24. | 8 | 8 | Iodoform and ol. morrhuæ. | |
| Oct. 10. | 8 | 10 | Easton's syrup. | Diarrhœa. |
| Dec. 7. | 8 | 11 | | |
| 1886—Jan. 4. | 8 | 9 | Iodoform resumed. | |
| Feb. 8. | 8 | 11 | | |
| Mar. 22. | 8 | 11 | Pil. omitted. | Sight misty. |
| May 31. | 8 | 7 | Fellows' syrup. | No bacilli found. |
| July 5. | 8 | 5 | Iodoform resumed. | |
| Aug. 16. | 8 | 10 | | |
| Sep. 13. | 8 | 10 | | |
| Oct. 11. | 8 | 9 | Iodol gr. j. in pill, t. d. s. | |
| Nov. 8. | 8 | 11 | Iodol with terebene. | |
| Dec. 6. | 9 | 0 | | |
| 1887—Jan. 17. | 9 | 1 | | |
| June 6. | 9 | 1 | Iodol discontinued. | Giddy, drowsy. |
| July 4. | 8 | 9 | Subcutaneous injections of eucalyptus, iodol and vaseline, once or twice a week. | Some irritation followed. |
| Aug. 29. | 8 | 8 | | |
| Sep. 22. | 8 | 7 | | |
| Oct. 3. | 8 | 8 | Creasote in glycerine, *one minim.* | Headache, giddiness. |
| Oct. 17. | 8 | 10 | | |
| Nov. 28. | 8 | 12 | | |
| 1888—Jan. 9. | 9 | 0 | | |
| Jan. 23. | 9 | 2 | | |
| Mar. 22. | 9 | 1 | Creasote increased, ♏iv. t. d. s. | |
| Apl. 5. | | | Subcutaneous injections of camphor carbolate, once or twice a week, ♏xx., for six weeks. | |
| Apl. 22. | 8 | 8 | | |
| May 3. | 8 | 8 | | |
| May 24. | 8 | 12 | Creasote ♏vij. t. d. s. | |
| Sep. 17. | | | | No bacilli in sputum. |

This patient's general condition continues good: he has never given up his regular occupation, excepting when an in-patient in 1885. He has now no symptoms of active disease in the lung, but the physical signs indicate fibrous condensation of the part originally affected. No bacilli have been detected in the sputum since the series of injections into the lung in 1885; and although he has had threatenings of active mischief, there has been no decided evidence of any progress or extension of the disease during the three years which have elapsed.

Various other solutions of iodoform and iodol were used hypodermically, but in each case there was more or less evidence of irritation in the cellular tissue, therefore no injection into lung was made with either; and in consequence of the unsatisfactory result of these early injections, the practice was discontinued for a time, until some fluid could be found which would not irritate the cellular tissue, and which might be powerful enough to modify germ development if injected into the lung.

I was reluctant to give up the idea that iodoform or iodol in some newer form of solution might answer our purpose; but after numerous demonstrations of local subcutaneous irritation from their use, I became convinced that it could not be a safe and harmless proceeding to inject them into the lung. On looking about to discover a substitute for them, and finding that carbolic acid, diluted with vaseline oil, was giving good results for hypodermic use in the hands of Meunier, it occurred to me that the saturated solution of carbolic acid in camphor, suggested by Dr. Cochran,* of Shomond, Wis., might answer the purpose. The 95 % solution of carbolic acid dissolves about three times its weight of camphor; the resultant being a thin, clear, oleaginous mixture, having a strong odour of camphor and a faint one of phenol. This fluid could be injected into the skin with impunity, and might be used as a solvent for iodoform (40 grs. to ℥j.).

* *Therapeutic Gazette*, Dec., 1887.

On testing the fluid, we soon found it possible to inject quantities of 30 to 40 minims beneath the skin without subsequent damage to the tissue injected; and after a few minutes' stinging, all evidences of irritation subsided. The fact that the carbolic acid was diluted with a substance which is itself an equally powerful parasiticide obviously gave the camphor a great superiority over the liquid vaseline, and it was assumed that the compound, although not irritating, must be a powerful germ-poison. Numerous cases were treated with this fluid, in all hypodermically at first, in many by intra-pulmonary injection afterwards. Two such cases are reported in detail in the September number of the *Bristol Medico-Chirurgical Journal*, page 185.

Solutions of iodoform, iodol, and creasote in the carbolic camphor were used hypodermically; but as they always gave rise to some local irritation, were not employed for injection into lung.

The result of these cases, and many others, appeared to show that in the camphor carbolate we had a fluid which might be injected into cellular tissue, and also into lung, without giving rise to local irritation or toxic symptoms: it caused little or no cough, and the patients continued to improve steadily under its use. In one case there had been evidence of temporary pneumothorax, which soon passed off, leaving the lung apparently as good as before: this was the only untoward event arising from it, and we had got to look upon this fluid as non-irritating, and its injection into the lung as a perfectly harmless proceeding, when the following case occurred:

Francis G., æt. 62. No family history of phthisis. An attack of bronchitis last winter, and a succession of colds, followed by continued cough, with expectoration and wasting. On June 30th coughed up some blood, and again on several occasions during the two following days. On admission, July 3rd, was found to be a spare, but not emaciated man, with good colour and clubbed fingers. Complains of paroxysmal cough, and brings up bright-red blood frequently. Right upper lobe dull, front and back, with weak breath-sounds and sibilant rhonchi. Doubtful sounds at left apex also.

July 5. Hæmoptysis had ceased, but temp. was 102°.

July 6. Sputum purulent, nummulated, with bacilli in small number. Temp., evening, 99.8°

July 18. Subcutaneous injections of biniodide of mercury, watery solution of 1 in 1000, given in left forearm, ♏xxx.

July 19. No irritation followed; injections continued daily.

July 24. Hydrarg. biniodid, with iodide of potassium, dissolved in camphor carbolate, 1 in 500, injected into forearm.

July 29. Injections had been repeated on 25th, 26th, 27th, and both arms were much swollen in consequence. Injection now repeated in leg.

Aug. 4. Injections discontinued because of much swelling in leg. Temp. normal; general condition good. No more hæmoptysis.

Aug. 7. Camphor carbolate without mercurial, ♏ xxx, injected into buttock, and continued daily.

Aug. 11. No irritation from the injections. Large patch of dulness at right upper lobe, none in left; moist sounds, voice-sounds exaggerated, below and above right clavicle and in supra-spinous region. An injection into apex of right lung, immediately below clavicle, one drachm of camphor carbolate being injected to a depth of nearly two inches, was immediately followed by much coughing, and patient tasted the camphor in his mouth: shortly he complained of irritation in the throat, and expectorated much frothy mucus smelling strongly of camphor; afterwards there was difficulty in breathing and aphonia, with towards evening copious purulent expectoration.

Aug. 12. Difficulty in breathing had continued, and bronchial rales were heard now both sides of chest; the signs of bronchitis increased rapidly, breathing became more and more laboured, and he died at 2 a.m. on the 13th, about 40 hours after the injection.

At the *post mortem* examination a cavity was found in the apex of the right lung: it was superficial, as large as a small hen's-egg, and contained some grumous fluid. There was extensive tuberculous infiltration of the whole of the right upper lobe, but none elsewhere. The bronchial mucous membrane was much injected and thickened, and on section of lung thick muco-pus exuded from the bronchial tubes of both sides. It was clear that the fluid injected had gone at once into the cavity, and, finding its way along the bronchus, had set up irritation of the mucous membrane along the whole track. Such an attack of acute suppurative bronchitis, coming on immediately after an injection into the lung substance, shows that the fluid, although harmless in the connective tissues, is a powerful irritant to the bronchial mucous membrane, and therefore inadmissible for future use, inasmuch as, although it is not intended that the fluid shall find its way into a cavity or a branch of the bronchial tree, it is impossible to say when this may be the case wherever on the chest-surface the needle may be introduced. I have been extremely reluctant to give up the use of a fluid which was giving such excellent results; but the occurrence of one such accident as that described, and the impossibility of guarding against the recurrence of similar results, compelled me to yield to the evidence of facts, and await the finding of a more satisfactory fluid before attempting further intra-pulmonary injections.

It was thought that the new substance, Sozoiodol, an acid sodium salt of iodparaphenolsulphonic acid, might have been of use; but, on injecting an aqueous solution under the skin, it was found to give rise to much irritation, and was discontinued. This had been the case also with boro-glyceride in glycerine, the sulpho-carbolates with and without creasote, oleic acid, solutions of mercurials, corrosive sublimate with ether in vaseline oil, and some others. Lépine * tried intra-pulmonary injections of 2 per cent. solutions of creasote in alcohol; and Dr. Leo Rosenbusch, of Lemberg, reports † a series of cases treated by injection of a 3 per cent. solution of creasote in almond oil. The injections were made by means of the Pravaz syringe, one-half syringeful being injected into each apex every second or third day.

Cough became less violent, temperature fell, sputum diminished, dyspnœa disappeared, and perspiration ceased. General condition and physical signs both improved. The creasote should be of vegetable origin.

One cannot but think that these cases illustrate the fallacies of the clinical method: the quantity of creasote injected must have been too small to give a decided result. On numerous occasions we have injected creasote solutions into cellular tissue, and always with the production of much irritation, so much so that we have not ventured to inject these solutions into the lung. An adequate dose will be certain to irritate: if no irritation occurs, the dose will be too little to be of service.

* *Medical Recorder*, March 20, 1888. † *New York Med. Record*, June 2, 1888.

A fluid, which, has been much used by Dr. White* of New York, has the following composition:

| | |
|---|---|
| ℞ Atropiæ ... ... ... ... ... | gr. $\frac{1}{3}$. |
| Morph. Sulphat. ... ... ... | gr. $\frac{1}{4}$. |
| Tinct. Iodinii ... ... ... ... | ʒiij. |
| Acid. Carbolic. (pur.) ... ... | ♏ 20. |
| Glycerin. ... ... ... ... ... | ʒjss. |
| Aquæ ... ... ... ... ... | ℥ij. |

Misce. Fifteen to thirty mimins for one injection.

This fluid we have found to be non-irritating to cellular tissue, and therefore admissible for intra-pulmonary use; it might be safely used for injection into lung, and the experience of Beverley Robinson† and Dr. White has been in favour of some such preparation of iodine, with or without carbolic acid. The various solutions of iodoform have always appeared to me superior to any other form of iodine administration, and hence the tincture has not as yet been submitted to any considerable test: the more so as the biniodide of mercury contains the germicide powers of the iodine to a much greater degree, and appeared likely to give more satisfactory results.

Recent observations with regard to the treatment of syphilis by intra-muscular injections of mercurial salts‡ appear to show that the most powerful of all germicides, the biniodide and the perchloride of mercury, may be used with impunity; and the cases treated by Miquel and Rueff§, by means of pulverisations of 1 in 1000 of the biniodide, appear to have been benefited by this mode of treatment. The solution used by Bloxam was as follows:

| | |
|---|---|
| Hydrarg. Perchlorid. ... ... | grs. 32. |
| Ammon. Chloridi... ... ... | grs. 10. |
| Aquæ ... ... ... ... ... | ℥ij. Misce. |

* *New York Med. Record*, May 22, 1888. † *New York Med. Record*, Jan. 10, 1885.

‡ J. Astley Bloxam, *Lancet*, April 28, 1888.

§ *Tuberculose Pulmonaire*. Paris. Masson, 1888.

Ten minims, containing one-third of grain of the salt, injected once a week, gave good results in syphilis.

A solution used by Yvon, which gave rise to no local reaction, is made up as follows :

| | | | | |
|---|---|---|---|---|
| Hydrarg. Biniodid. | ... ... | | gr. j. | |
| Potass. Iodid. | ... ... ... | | gr. j. | |
| Sodic Phosphat. | ... ... ... ... | | grs. ij | |
| Aq. destillat. | ... ... | ad | ♏ 50 | Misce. |

Five minims = $\frac{1}{10}$th of a grain.

The biniodide of mercury is said to be the most powerful antiseptic substance known, and the following summary of a table given by Miquel and Rueff* is worthy of study:

| | | | | | | |
|---|---|---|---|---|---|---|
| Biniodide of Mercury— | Solution | aseptic | at | 1 | in | 40,000 |
| Iodide of Silver ... ... | ,, | ,, | ,, | 1 | ,, | 33,000 |
| Peroxide of Hydrogen | ,, | ,, | ,, | 1 | ,, | 20,000 |
| Bichloride of Mercury.. | ,, | ,, | ,, | 1 | ,, | 15,000 |
| Iodine ... ... ... ... ... | ,, | ,, | ,, | 1 | ,, | 4,000 |
| Iodoform ... ... ... ... | ,, | ,, | ,, | 1 | ,, | 1,660 |
| Sulphate of Copper ... | ,, | ,, | ,, | 1 | ,, | 1,100 |
| Acid Salicylic ... ... | ,, | ,, | ,, | 1 | ,, | 1,000 |
| Carbolic Acid ... ... | ,, | ,, | ,, | 1 | ,, | 300 |
| Tannin ... ... ... ... ... | ,, | ,, | ,, | 1 | ,, | 210 |
| Arsenious Acid ... ... | ,, | ,, | ,, | 1 | ,, | 165 |
| Soda Salicylate ... ... | ,, | ,, | ,, | 1 | ,, | 100 |
| Sulphate of Iron ... ... | ,, | ,, | ,, | 1 | ,, | 90 |
| Alcohol ... ... ... ... ... | ,, | ,, | ,, | 1 | ,, | 10 |
| Hyposulphite of Soda... | ,, | ,, | ,, | 1 | ,, | 3·7 |

The biniodide of mercury is therefore the substance which seems most likely to be effectual for the purpose in view, to act as a local germicide on a diseased patch. If a satisfactory solution can be found which may be injected into the lung without risk of injury, and in sufficient quantity, it seems likely that the local germicide action may be efficient and without risk from general blood contamination.

* *Tub. Pulm.* Paris. Masson, 1888.

On the other hand, it should be noted that the mercurial salts do not take so high a relative position in the lists drawn up by Villemin,* who classifies various substances in accordance with their effects on the bacillus of tubercle. He finds that the following substances do not at all interfere with the growth of the bacillus :—Phenic acid, salicylic acid, biborate and benzoate of soda, aniline oil, &c.; and that in the second category may be classed those which give less prosperous and slower cultures, comprising—Acetanilide, arseniate of soda, biniodide of mercury, camphor, eucalyptol, quinine, resorcine, terpine, thymol, &c.

He observes that clinical experience shows that the mercurial biniodide, acid benzoic, acid salicylic, and borax are medicaments which destroy the germs of the air in wounds and elsewhere, but he affirms that their efficacy is *nil* with regard to the one particular species, the bacillus of tubercle.

The third last contains those substances which notably retard the development of the bacillus in the agar tubes, and comprises:—Arsenious acid, boracic acid, alcohol, chloroform, creasote, ether, iodoform, phenate of soda, salol, &c.

The fourth list comprises a small number of substances which completely sterilise: they are—Acid hydrofluoric, fluosilicate of iron, potassium, and sodium, naphthol $\alpha$, naphthol $\beta$, and sulphate of copper.

He then remarks that the bacillus of tubercle has a very considerable vital power of resistance: that it is possible to retard its development, so that its reproduction is accomplished with great slowness; but it can only with difficulty be arrested completely.

* *Etudes sur la Tuberculose*, Tome II. Paris. 1888.

The substances mentioned as having the greatest power in this respect are being tested, and some of them have been well reported on as giving good results clinically. Hydrofluoric acid has been employed by Hérard, Lépine, Paliard, and others; but although good results have been reported from the inhalation of the diluted acid, the evidence is conflicting and as yet doubtful. Tannin has been well reported on by Caccherelli,* who says that, although from the antiseptic point of view it is inferior to many other drugs, from the anti-tuberculous point of view it has been found to be very useful and more efficacious than iodoform. Sulphate of copper had also its advocate, Prof. Luton, at the recent Congress in Paris; but it has yet to show its superiority over other substances.

Many of these drugs have been used by Dr. Cornet,† in his experiments at the Berlin Hygienic Institute; and it is disappointing to find how little success he obtained in his endeavours to render the tissues of animals unsuitable as a cultivating medium for tubercle bacilli. Tannin, sulphuretted hydrogen, bichloride of mercury, and creasote were all given in large doses in relation to the body-weight of the animal, and given by injection. Every animal died, from the inoculated disease, there being no hindrance to the development of the bacilli by any one of the remedies given. Further, it was found that infected guinea-pigs on being sent to Davos-platz, while others were kept in Berlin, all died about the same time.

In spite of the doubts thrown by Villemin on the efficacy of the biniodide of mercury, we have treated

* *Etudes sur la Tuberculose,* Tome II. Paris. 1888.

† *Lancet,* May 19, 1888.

some cases by the hypodermic and intra-pulmonary injections of this mercurial salt, and have found that it may easily be injected, day by day, in doses of $\frac{1}{10}$th of a grain, without giving rise to ptyalism; and when injected into the lung (gr. $\frac{1}{5}$) gave only temporary discomfort, with no rise of temperature. The following case has recently been under treatment:

Isaac T., æt. 55. Cough for six months, hæmoptysis on several occasions, getting thin, lost from 10st. 10lb. to 8st. 11lb., clubbed finger-tips, purulent expectoration with a few bacilli, patch of dulness in left upper lobe. Was injected daily with camphor carbolate, in drachm doses, for 17 days; then with iodine solution (White's), 15 to 30 minims daily for 10 days; then with hydrarg. biniodide, 10 minims (containing one-fifth of a grain), for 11 days: these injections were all hypodermic, usually in the buttock, and they gave rise to no irritation or discomfort. Three injections of the mercurial biniodide were then made into the condensed left upper lobe at intervals of one week; twenty minims was the quantity used on each occasion, representing $\frac{2}{5}$ths of a grain of the salt. The first gave rise to no cough, no pain, and no evidence of irritation at any time; the second, given in the axilla, gave some considerable pain, lasting for an hour, but no cough and no other evidence of irritation; the third, given on October 3rd, in the first interspace to a depth of two inches, caused no pain and no subsequent discomfort. Thirty-eight subcutaneous injections were administered in about as many days, the fluids used amounting to two ounces of the camphor carbolate, half an ounce of Dr. White's iodine solution, and quarter of an ounce of the biniodide of mercury, containing over two grains in all. Subsequently three injections were made into the upper lobe of left lung, amounting to sixty minims, containing six-fifths of a grain of the biniodide.

The patient's temperature continued subnormal during the whole course of treatment, and the weight increased from 8 st. 11 lbs. to 9 st. 8 lbs. The expectoration gradually diminished, and contained only a very small number of bacilli; at last none could be obtained for examination. There was no cough, and the patient appeared to be in fairly good health, although there was of necessity still evidence of condensation in the left upper lobe.

This case, and others in which a watery solution of biniodide of mercury has been injected, quite encourage the view that this drug, whilst it is one of the most potent germicides, is at the same time one of the least irritating for intra-pulmonary use. I shall therefore prefer this to any other fluid we have tried, until there arises evidence in favour of something better.

It will be seen that there is much to be said both against and in favour of lung injections. The following list of unsatisfactory results suffices to show that the method is not devoid of risks:

Acute suppurative bronchitis, fatal in 48 hours.

Acute pleuritis, two cases, speedily recovering.

Pneumothorax, temporary and harmless. (A similar case occurred to Dr. A. Ransome).

Intense pleuritic pain, lasting for hours, and requiring full doses of morphia.

Violent fits of coughing. (Iodoform poisoning, and hæmorrhage from lung, were observed by Dr. A. Ransome).

In the presence of such results as these, we cannot but hesitate before submitting a patient to a process which may entail serious consequences; but, on the other hand, it seems likely that all these accidents may be avoided by increased experience and improved methods. In only one of the above was any real harm done to the patient: even in the case of pneumothorax the only discomfort was dyspnœa on exertion lasting a few days and the pleuritis cases speedily recovered.

We have learnt that for safety it is necessary that the fluid should pass into the lung-tissue itself; otherwise, if it reaches the pleura only, it may give much pain and pleuritis; if it reaches a bronchus or a cavity, it may give rise to bronchitis: in either case, if the fluid be injected slowly it will be possible to alter the position of the needle before injecting the whole quantity. Again, the effect of the injection must vary immensely with the qualities of the fluid injected and the quantity employed; as yet we are only on the threshold of possibilities in this direction, and are not even in a position to answer the questions, "What fluid is the best?" and "How much is to be introduced?" The results I have given may conduce to a more satisfactory solution to these questions. I have shown what fluids may not be injected without some risk. Possibly increased experience of the biniodide of mercury may confirm our present opinion of its safety and may establish its utility.

As regards the decision of what cases are suitable for injection, it seems clear that, in accordance with the principle of local treatment, we should exclude those in which both lungs are implicated, and also those in which the disease, although limited to one lung, is widely distributed throughout it. If the case is hopelessly beyond a reasonable probability of benefit from injection, I should myself prefer not to bring the method into discredit by adopting it uselessly. We are therefore limited to cases where the mischief may still be looked upon as local, but these are the cases in which very active treatment does not seem necessary to the patient, who is unwilling to submit to active interference when not compelled to it by active symptoms. The principle I have adopted has been to adopt milder methods first, and only fall back upon the intra-pulmonary injections when other kinds of treatment are failing to arrest the disease.

*c. Pulverisations and Inhalations.*—These have been much in vogue of late. The recent debate on the subject of inhalations at Glasgow shows how divided opinions are as to their utility. I do not propose to enter into this question, but must suffice by remarking that, although clinically they appear to be of service, and more especially when given continuously, as advocated by Cohen and Burney Yeo, I have not been able to trace any definite curative result to their undivided use.

The inhalation of diluted hydrofluoric acid, first advocated by Bergeron, has recently been tested on rabbits by Grancher and Chautard of Paris, who found that inoculated rabbits died at the same time whether treated in this way or not.*

The pulverisations of biniodide of mercury employed by Miquel and Rueff appear to have been useful. They publish a series of 27 cases, and claim for the treatment the following general effects: increase of appetite and weight; diminution or disappearance of the pyrexia, night-sweats, and expectoration.

In five cases where the bacilli had disappeared from the sputum the pulverisations had been continued uninterruptedly for eight to ten months. It remains yet to

* *Therapeutic Gazette*, July, 1888.

be proved whether this method is likely to meet with general acceptance and to give good results.

I have spoken hitherto of the offensive method, aiming at the destruction of the tubercle bacillus and of the other bacteria which have been recently shown to accompany it in tuberculous lungs. Now it is generally admitted that as yet the defensive methods, by which the body is fortified to withstand the bacillary attack, have given the most satisfactory results; and it is believed by many that general hygienic surroundings, including diet, good air, and exercise, are of primary importance, leaving medicinal treatment altogether in the background. There cannot be two opinions as to the necessity for careful attention to all the conditions which may tend to improve the nutrition of the tissues; even if an attempt is made to attack the germs, every other effort to improve nutrition is of great importance, and should not be set aside in favour either of pulveriser, inhaler, or injection syringe. The only point to which I wish to give emphasis is the desirability of doing all which can be done by an entire alteration in the surroundings and conditions of life, such as a change of climate entails. Time prevents me from entering fully into this question, but we cannot close our eyes to the fact that a great diminution of atmospheric pressure, and pure, absolutely dry, and comparatively aseptic air, such as are to be found at Davos and other similar health resorts, have accomplished in individual cases what everything else has hitherto failed to do.

One other point must not be passed over in silence: the method of forced alimentation by means of the stomach-tube, called by the French "lavage and gavage." We all know how important is the function of digestion, and how great an influence anorexia and other gastric difficulties have in counteracting all our efforts. If the nutritive processes be not active, the patient's condition is well-nigh hopeless: in the battle between the tissue protoplasm and the bacilli, the latter are certain to win in cases where the digestive powers are feeble and cannot be restored. In such cases as these, daily washing of the stomach, and the introduction of food in excess of that which could be swallowed, have given good results; but of this method I have had no experience, and therefore cannot speak with any authority.

Gentlemen,—It only remains for me now to thank you for the honour you have conferred in placing me in this Chair, and for the patient hearing you have given to my theme. My predecessor, quoting his favourite author, might remind me that "it is better to be brief than tedious." I fear I have incurred some risk of the latter, and will not take up more of your time by any eulogy of the Society, or by any details of its history. The minute-book bears witness to its prosperity in the past, and its quarterly journal is a visible demonstration of its utility in the present. As regards its future, I trust that no cloud will arise to impede its progress, but that we shall continue to advance and expand until in the new Medical School of the not distant future, we shall have a local habitation, with a library, and be even more worthy of support and sympathy from all the medical fraternity of the West than as yet we can claim to be.

